# Exogenous melatonin ameliorates embryo–maternal cross-talk in early pregnancy in sheep

**DOI:** 10.1530/REP-24-0172

**Published:** 2024-10-03

**Authors:** Irene Viola, Cecilia Sosa, Paolo Accornero, Isabella Manenti, Francisco Canto, Silvia Miretti, José Alfonso Abecia, Paola Toschi

**Affiliations:** 1 Department of Veterinary Sciences, University of Turin, Grugliasco, Torino, Italy; 2 Department of Biochemistry and Cell and Molecular Biology, Faculty of Veterinary Medicine, University of Zaragoza, Zaragoza, Spain; 3 Environmental Science Research Institute (IUCA), Faculty of Veterinary Medicine, University of Zaragoza, Zaragoza, Spain

## Abstract

**In brief:**

Melatonin plays a crucial role in enhancing reproductive performance in small ruminants. This paper reveals the effects of exogenous melatonin on the placental and endometrial rearrangement in early pregnancy in sheep.

**Abstract:**

Early pregnancy losses cause 25% of pregnancy failures in small ruminants because of asynchrony between conceptus and uterine signals. In this context, melatonin plays a crucial role in sheep reproductive dynamics, but little is known about its effects during the peri-implantation period. We hypothesized that melatonin supports embryo implantation by modulating the uterine microenvironment. This study aimed to assess the effects of exogenous melatonin on the endometrial and early placental rearrangement. Ten multiparous ewes either did (MEL, *n* = 5) or did not (CTR, *n* = 5) receive a subcutaneous melatonin implant (18 mg) 50 days before a synchronized mating. On day 21 of pregnancy, the sheep were euthanized. MEL ewes exhibited a higher prolificity rate (2.8 vs 2.0 embryos/ewe) and plasma progesterone levels (3.84 vs 2.96 ng/mL, *P* < 0.05) than did CTR ewes. Groups did not differ significantly in embryo crown-rump length. MEL placentas had significantly (*P* < 0.001) more binucleated trophoblast cells in the chorion region, and ovine placental lactogen expression was significantly (*P* < 0.05) more strongly upregulated than in CTR. Exogenous melatonin increased significantly (*P* < 0.05) gene expression of angiogenic factors *(VEGFA, VEGFR1, IGF1R)*, *IFNAR2*, and *PR* in the caruncular endometrium. Expression of the MT2 receptor in the endometrium and placenta was significantly (*P* < 0.05) higher in the MEL group. These results indicate that melatonin implants acted differentially on uterine and placental rearrangement. Melatonin increases differentiation in the placenta and induces changes that could promote vessel maturation in the endometrium, suggesting that it enhances the uterine microenvironment in the early stage of pregnancy in sheep.

## Introduction

Management of reproduction in ruminants has improved to ensure specific production standards; however, among reproductive disorders, early pregnancy losses cause 25% of gestation failures in small ruminants and up to 40% in cattle (Diskin & Morris 2008, De la Concha-Bermejillo & Romano, 2021). Therefore, studying reproductive dynamics in the early stages of pregnancy is important to improve the reproductive health of sheep.

Melatonin (N-acetyl-5-methoxy tryptamine) is a hormone that plays a fundamental role in the regulation of reproductive seasonality in sheep. Subcutaneous melatonin implants can regulate the length of the ovine breeding season, which increases animal fertility and prolificity. Apparently, that is because of the protective role of melatonin, which helps to maintain a uterine environment that promotes fetal growth and survival (Abecia *et al.* 2018). Melatonin produced by the mother can cross the placenta unaltered (Torres-Farfan *et al.* 2008, Eifert *et al.* 2015) and can improve embryo quality by reducing oxidative stress, enhancing corpus luteum competence, and modulating the mother’s mechanism for recognizing pregnancy in sheep (Abecia *et al.* 2008). Melatonin acts by binding to the MT1 and MT2 receptors, which are expressed in the ovine reproductive system (González-Arto *et al.* 2017, Abecia *et al.* 2018). The expression of melatonin receptors occurs in the ovine endometrium throughout the estrous cycle (Sosa *et al.* 2023). Melatonin receptors are present in the trophoblast of ovine blastocysts, which suggests that melatonin might play a role in placental development (Casao *et al.* 2019); however, most research into the function of melatonin in placentation in ruminants has centered on the latter stages of gestation (McCarty *et al.* 2018, Trotta *et al.* 2021). Furthermore, most research has involved undernourished sheep in assessing the recovery power of melatonin on the reproductive system and pregnancy because of melatonin’s putative antioxidant and free-radical-scavenging effects (Lemley *et al.* 2012, Vázquez *et al.* 2013, Cosso *et al.* 2021). Little is known about the effects of melatonin implants in the early stages of pregnancy in sheep. Melatonin alters the physiology and survival of the conceptus; therefore, we suspected that melatonin promotes embryo implantation by simultaneously acting on the endometrial and placental rearrangement. The objective of this study was to explore whether and how exogenous melatonin improves the uterine microenvironment to support normal early placental development, allowing pregnancy proceeding in sheep. To that end, we focused on conceptus (fetus and placenta) morphology and hormonal and molecular changes that occur in the uterus and placenta following melatonin treatment. In particular, we selected a panel of genes that regulate vital mechanisms in the peri-implantation window such as cell growth and adhesion, tissue vascularization, and hormonal trafficking.

## Materials and methods

### Ethical approval

The flock was housed at the experimental farm of the University of Zaragoza, Spain (41°40′ N 0°53′ W). The experiment followed a protocol (PI47/21) that was approved by the Ethics Committee for Animal Experiments at the University of Zaragoza and was in accordance with the requirements of the European Union for Scientific Procedure Establishments.

### Animal management and melatonin treatment

In late February, 50 days before synchronized estrus, 20 multiparous Rasa Aragonesa ewes (mean body weight ± s.d.: 58 ± 4 kg; BCS: 2.75 ± 0.40) were selected from the experimental sheep flock at the University of Zaragoza, which either did (MEL group, *n* = 10) or did not (CTR group, *n* = 10) receive a single subcutaneous melatonin implant (18 mg; CEVA Salud Animal, Barcelona, Spain) at the base of the left ear. Two weeks after implantation, intravaginal progestagen sponges (fluorogestone acetate 30 mg, Sincropart, CEVA Salud Animal) were inserted for 12 days. At pessary withdrawal, 480 IU eCG (Sincropart, CEVA Salud Animal) was administered, and five rams of proven fertility were introduced into the flock 48 h later and remained for 2 days. Twenty-one days after the onset of mating, the ewes were euthanized (T-61, MSD Animal Health, Salamanca, Spain; 4–6 mL/50 kg live weight), and ten pregnant ewes (5 from each group) were randomly selected for conceptus and endometrium sampling. Blood samples were collected from the jugular vein using a heparin tube vacutainer on the day of implant insertion, sponge removal, and the last day of the experiment.

### Sample collection

The conceptus was extracted through an incision in the uterine horns and, to assess embryo vitality, viewed under a stereomicroscope. A microsurgical slicer and tweezer were used to separate the entire placenta from the embryo. To measure crown-rump length (Ptak *et al.* 2013), photos of each embryo, including a reference meter, were analyzed with the image processing software ImageJ Fiji 1.53s (NIH; https://imagej.nih.gov/ij/). Thereafter, the embryos were fixed in 4% formaldehyde at 4°C overnight before being stored in 70% ethanol at room temperature (RT) for histological examination.

Placenta samples were snap-frozen in liquid nitrogen and stored at −80°C for molecular analysis or fixed in 4% formaldehyde and stored in 70% ethanol for histological and histochemical analyses. After the conceptus was removed, the uterus was placed on ice, and samples of caruncular and intercaruncular endometrium were dissected from the middle third of the placenta-bearing uterine horn, snap-frozen in liquid nitrogen and stored at −80°C.

### Progesterone analysis

Plasma was immediately separated by centrifugation (2000 × ***g*** for 10 min at 4°C) and stored at −20°C. Extraction of progesterone (P4) from plasma was performed following modifications of the DetectX® Steroid Liquid Sample Extraction protocol (Arbor Assays) per Viola *et al.* (2023). Hormone concentrations of day 21 samples were quantified based on a P4 ELISA kit (DRG Diagnostics GmbH) according to the manufacturer’s instructions. P4 concentrations were expressed as ng/mL.

### Histological procedure analysis

Fixed samples (placentas and embryos) were dehydrated in ethanol solutions in a range of concentrations (80%, 90%, 96%, and 100%) and cleared in a xylene mixture, 3 min for each step, following Kaufman (1995). Thereafter, placentas and fetuses were embedded in Paraplast. Serial sections (thickness = 5 μm for embryos and 7 μm for placentas) obtained by a microtome (Leica RM2155) were subjected to hematoxylin-and-eosin staining. Before staining, the sections were dewaxed in xylene, rehydrated in a series of ethanol solutions of decreasing concentrations, and washed in running tap water. The stained sections were cover-slipped using DPX mountant (Sigma, 06522) for histology. A Nikon Eclipse Ti2 High-Content Screening microscope was used to take photos. The proportion (%) of binucleated cells was calculated based on 15 randomly selected fields containing at least 500 cells.

### Immunochemistry

Placenta sections were placed on Superfrost microscope slides (Thermo Fisher Scientific, 10149870), dewaxed in xylene (twice for 3 min), rehydrated in a series of ethanol solutions of decreasing concentrations (100%, 96%, 90%, and 80%) for 1 min each, and permeabilized for 30 min in TBS (Tris 0.1 M, NaCl 8 g, pH 7.6) and 0.05% Tween. After a 30-min incubation in a warm antigen retrieval solution (Dako, S1699), the slides were left to cool at RT. Non-specific antigen sites were blocked with 1% BSA at RT for 1 h. Subsequently, samples were incubated with alpha-smooth muscle actin antibody (aSMA, 1:200; Sigma-Aldrich, A5228) for 1 h at RT. After extensive washing with TBS, slides were incubated in the dark with Alexa Fluor 594 goat anti-mouse secondary antibody (1:500; Invitrogen, A32723) for 1 h at RT. Simultaneously, negative control and no-primary antibody control were processed similarly. The slides were again washed with TBS before DAPI (Thermo Scientific, D1306) was added for 10 min at RT for nuclei staining. Photos were taken by a Leica SP8 confocal fluorescent microscope (Leica Microsystems) at 40× magnification.

### Western blot

A sterile glass potter was used to extract protein from placenta samples. One gram of tissue was lysed for 5 min on ice in 1 mL lysis solution (10 mM Tris HCl pH 7.4, 150 mM NaCl, 1 mM EDTA, 1% Triton X-100, 0.1% SDS, 0.5% sodium deoxycholate, and 0.01% sodium azide), a protease inhibitor cocktail (1:100), 1 mM sodium orthovanadate, and 1 mM phenylmethylsulfonyl fluoride. The lysate was centrifuged at 4°C for 15 min at 15,000 × ***g***, and the amount of protein in the supernatants was quantified by DC Protein Assays (Bio-Rad Laboratories) following the protocol’s instructions. For western blotting, samples (25 μg of total protein) were resolved on a 12% polyacrylamide gel and transferred to 0.2 μm nitrocellulose blotting membranes (Amersham Protran Premium). Membranes were blocked at RT for 1 h in 10% BSA TBS (TBS–Tween, 10 mM Tris and 150 mM NaCl, pH 7.4, 0.1% Tween 20), then incubated overnight at 4°C with antibodies against alpha-tubulin (aTUB, 1:10,000, Sigma, T5168) or melatonin receptors (MEL-1A/B-R, 1:600, Santa Cruz Biotechnology, 398788). Membranes were washed in TBS–Tween and incubated at RT for 1 h with HRP-conjugated secondary antibody (1:15,000). Membranes were washed in TBS–Tween and incubated for 5 min at RT with Clarity Western ECL Substrate (Bio-Rad Laboratories). The proteins were visualized by exposing the membranes to an autoradiographic CL-XPosure Film (Thermo Fisher Scientific). Western blotting results were acquired with an EPSON Perfection V39 scanner. Densitometry analysis was performed by ImageJ Fiji 1.53s (NIH; https://imagej.nih.gov/ij/). Melatonin receptor expression was calculated relative to aTUB (reference control), and the HC11 cell line was used as a positive control (Presman *et al.* 2006). Immunoblotting was repeated twice on four samples.

### RNA purification and retrotranscription

Total RNA from placenta samples was extracted using a Maxwell RSC simplyRNA tissue kit (Promega). RNA quality and concentrations were measured by Nanodrop (Thermo Fisher), and 1 μg was reverse transcribed together with a non-reverse transcribed control (no-RT) using an iScript^TM^ cDNA Synthesis kit (Bio-Rad Laboratories). Endometrial RNA was extracted using an NZY Total RNA Isolation kit (NZYTech, Lisbon, Portugal) with DNAse I treatment. RNA purity and concentrations were measured with a Nanodrop (Thermo Fisher), and integrity was assessed by electrophoresis. A First-Strand cDNA Synthesis Kit (NZYTech) was used to reverse transcribe 1 μg total RNA to cDNA. Retrotranscription reactions were performed in an iCycler Thermal Cycler (Bio-Rad Laboratories).

### Gene expression

Gene expression analysis was performed on the following genes: melatonin receptor-2 (*MT2*, NM_001130938.1, 5′-CCCAGAGGGGTTGTTTGTCT-3′; 3′-TTCCCTGCGGAAGTTCTTGT-5′); progesterone receptor (*PR*, Z66555.1, 5′-GTCCCTAGCTCACAGCGTTT-3′; 3′-TGCCCGGGACTGGATAAATG-5′); interferon-alpha receptor-2 (*IFNAR2*, NM_001009342, 5′-ACATTCAGCAGGGTTCATAGCA-3′; 3′-TTTCTGTGGCTTTTCTGGTCTTC-5′); ovine placental lactogen (*oPL*, NM_001009309.4, 5′-AGCAACAACGGTGGCTAACT-3′; 3′-GCCATACTGTTCATCAAATCTGTT-5′); vascular endothelial growth factor-A (*VEGFA*, NM_001025110.1, 5′-AAACCTCACCAAAGCCAGCA-3′; 3′-GCCTCGGCTTGTCACATTTTT-5′); vascular endothelial growth factor receptor-1 (*VEGFR1*, uterus, XM_015098156.3, 5′-AGGACCTGAAGCTGTCTTGC-3′; 3′-GTTGCGTGGTCTGGTTGTTC-5′; placenta, AF488351.1, 5′-TGGATTTCAGGTGAGCTTGGA-3′; 3′-TCACCGTGCAAGACAGCTTC-5′); vascular endothelial growth factor receptor-2 (*VEGFR2*, NM_001278565.2, 5′-AGACAGAACCAAGTTAGCCCC-3′; 3′-TAGCCGCTTGTCTGGTTTGA-5′); insulin growth factor-2 (*IGF2*, uterus, NM_001009311.1, 5′-GGCTTCTACTTCAGCCGACC-3′; 3′-GGCACAGTAAGTCTCCAGCA-5′; placenta, 5′-TTCTTGCCTTCTTGGCCTTCG-3′; 3′-AAGCAACACTCTTCCACG-5′); insulin growth factor receptor-1 (*IGF1R*, uterus, AY162434.1, 5′-GGCTCAACCCAGGGAACTAC-3′; 3′-AGAAGAACACAGGCTCCGTC-5′; placenta, XM_027957015.2, 5′-GGACGGAGTACGCCG-3′; 3′-AGGGAGGGCGGGTTC-5′); angiopoietin-1 (*ANGPT1*, XM_004011787.5, 5′-GTGCAAATGTGCCCTCATGC-3′; 3′-TTTCCAAGGTTCTGTCCCGC-5′); angiopoietin-2 (*ANGPT2*, uterus, XM_004021671.5, 5′-TGGGTGGACGGTTATTCAGC-3′; 3′-GGGTTCCCGAATCCCACTTT-5′; placenta, XM_004021671.6, 5′-ATAGAAATAGGGACCAACC-3′; 3′-TTCTTATCTTGCAGTTTGC-5′); angiopoietin receptor (*TIE2*, AY288926.1, 5′-TTACCAGGTGGACATCTTTGC-3′; 3′-TTGGGCCATTCTCCTTTGG-5′); mucin-1 (*MUC1*, XM_027976040, 5′-CACCACTGCTGAGTTGGTGA-3′; 3′-AGGAAGGAAACTGGGCATCG-5′); and osteopontin (*OPN*, NM_001009224.1, 5′-ACCCTCCCGAGTAAGTCCAA-3′; 3′-TCAGGGGTTTCAGCATCGTC-5′).

Placenta cDNA samples were amplified by quantitative PCR (qPCR) in a CFX Connect real-time PCR detection system (Bio-Rad Laboratories) using the SsoAdvanced Universal SYBR Green Supermix (Bio-Rad Laboratories). Each run was performed in duplicate under the following conditions: 40 cycles of 94°C for 45 s, 58/60°C for 45 s, and 72°C for 1 min. Real-time PCR of endometrium was performed in a LightCycler 480 (Roche Diagnostics) using the NZYSpeedy qPCR Green master mix (NZYTech) and under the following amplification conditions: 2 min at 95°C, and 40 cycles of 5 s at 95°C, and 30 s at 60°C.

At the end of each run, dissociation curves were analyzed to confirm amplicon specificity and the absence of contamination or primer dimers. To avoid false-positive signals, negative controls (no sample) were included in each run. The relative expression of each gene was calculated based on the comparative threshold cycle method, normalized to the following housekeeping genes: ribosomal protein S9 (*RPLS9*, XM_027978859.3, 5′-CAAGTCCATCCACCATGCCC-3′; 3′-GACGGGATGTTCACCACCTG-5′) and ribosomal protein L32 (*RPL32*, XM_004018540.4, 5′-AAAATCAAGCGGAACTGGCG-3′; 3′-GGCATCAAGATCCTGGCCCTT-5′) for placenta; β-actin (*ACTB*, NM_001009784, 5′- CTCTTCCAGCCTTCCTTCCT-3′; 3′-GGGCAGTGATCTCTTTCTGC-5′) and glyceraldehyde-3-phosphate dehydrogenase (*GAPDH*, NM_001190390.1, 5′- GGTTGTCTCCTGCGACTTCA-3′; 3′-AAGTGGTCGTTGAGGGCAAT-5′) for endometrium.

### Statistical analysis

Shapiro–Wilk tests confirmed whether the data followed a normal distribution. The statistical significance of differences in endometrial transcript expression was assessed by a two-way ANOVA that included the main effects of melatonin treatment, endometrial region (caruncular or intercaruncular), and their interaction, followed by Fisher’s least significant difference tests to assess differences between groups. Gene expression data from CTR and MEL placentas and plasma P4 were assessed by nonparametric Mann–Whitney *U*-tests.

The significance of differences between groups in the proportion of cells that were binucleated was analyzed by Fisher’s exact test. Data are reported as the mean ± s.d. for embryo crown-rump length, or mean ± s.d. for P4 concentrations, binuclear cell counts, gene expression analysis, and western blot densitometry. Statistical differences were considered significant if *P* < 0.05.

## Results

### Ewes and embryos *in vivo*

**Figure 1:**
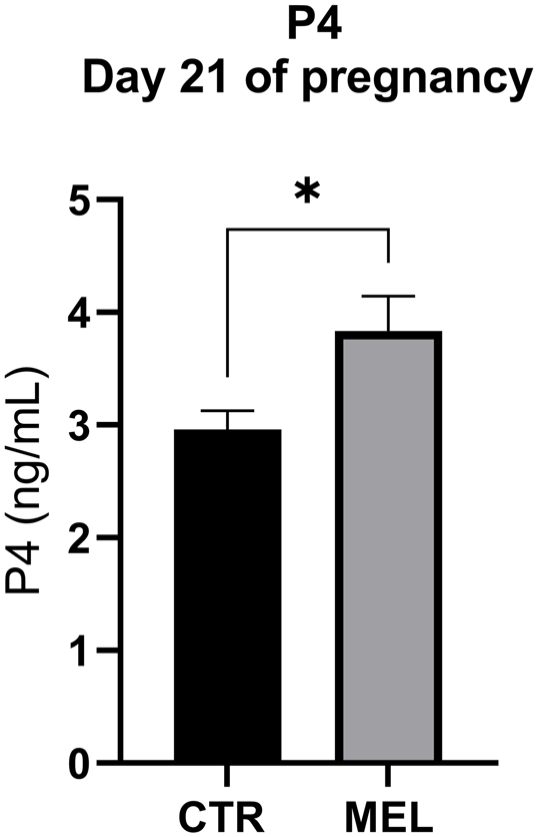
Plasma progesterone in pregnant ewes. Mean (±s.d.) plasma progesterone (P4) concentrations on day 21 of pregnancy in Rasa Aragonesa ewes that either did (MEL) or did not (CTR) receive a subcutaneous melatonin implant (*P < 0.05). Plasma samples were centrifuged and processed with diethyl ether at 80°C. P4 was extracted for evaporation by ultracentrifugation, and the concentration was measured by an ELISA kit and expressed as ng/mL. All analyses were repeated in duplicate. Melatonin implants increased the P4 level in plasma in pregnant ewes in the early stage of pregnancy.

**Figure 2:**
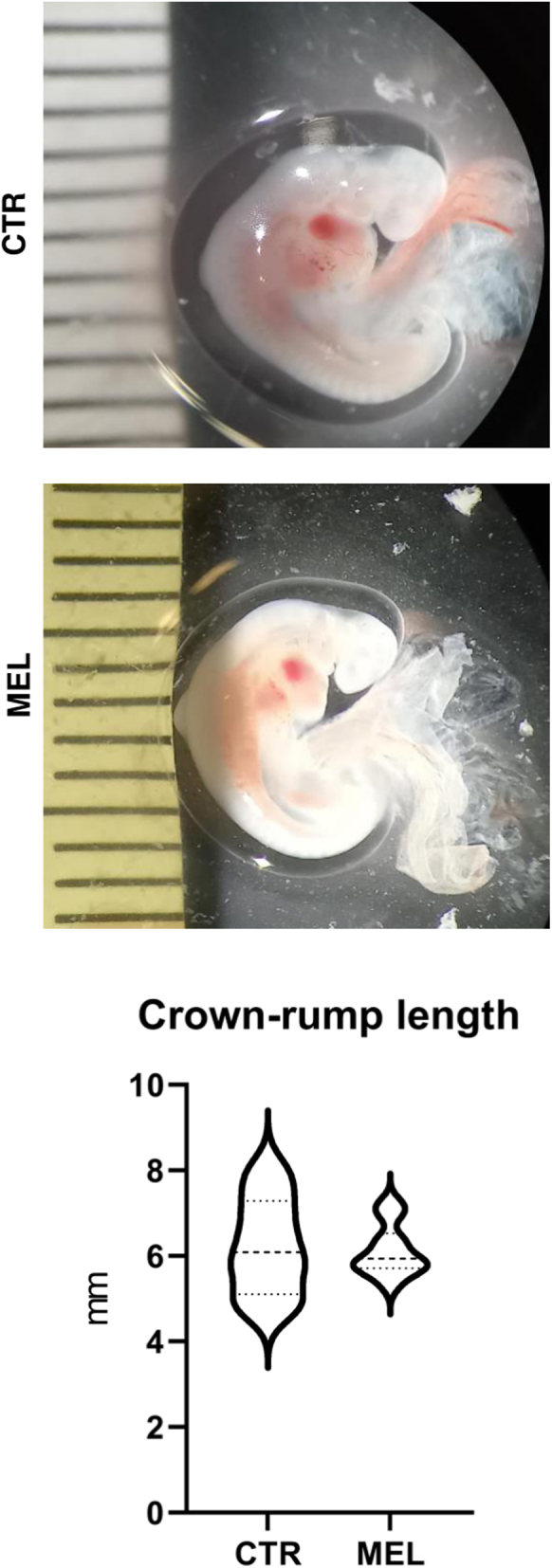
Embryo crown-rump. Mean (±s.d.) embryo crown-rump length measured immediately after uterus dissection on day 21 of pregnancy in Rasa Aragonesa ewes that either did (MEL) or did not (CTR) receive a melatonin implant. Embryos in the two groups appeared to be at the same stage in development (14–15 stage according to Carnegie classification (Butler and Juurlink 1987)) based on the measurement indicated in the violin plot (left). In addition, the distribution of embryos' crown-rump lengths was more restricted in the MEL group than it was in the CTR group.

MEL ewes had a higher potential litter size (2.0 vs 2.8 embryos/ewe, *P* < 0.05) and P4 plasma levels on day 21 (2.96 ± 0.45 vs 3.84 ± 0.81 ng/mL; *P* < 0.05) than did CTR ewes (Fig. 1). At the time of sampling, all embryos had heartbeats. The crown-rump length (6.2 ± 1.1 vs 6.1 ± 0.6 mm) did not differ significantly between groups; however, the distribution of the lengths of embryos appeared more restricted in the MEL group than it was in the CTR group (*σ*^2^ 1.32 vs 0.36) (Fig. 2).

### Endometrium gene expression profile

**Figure 3:**
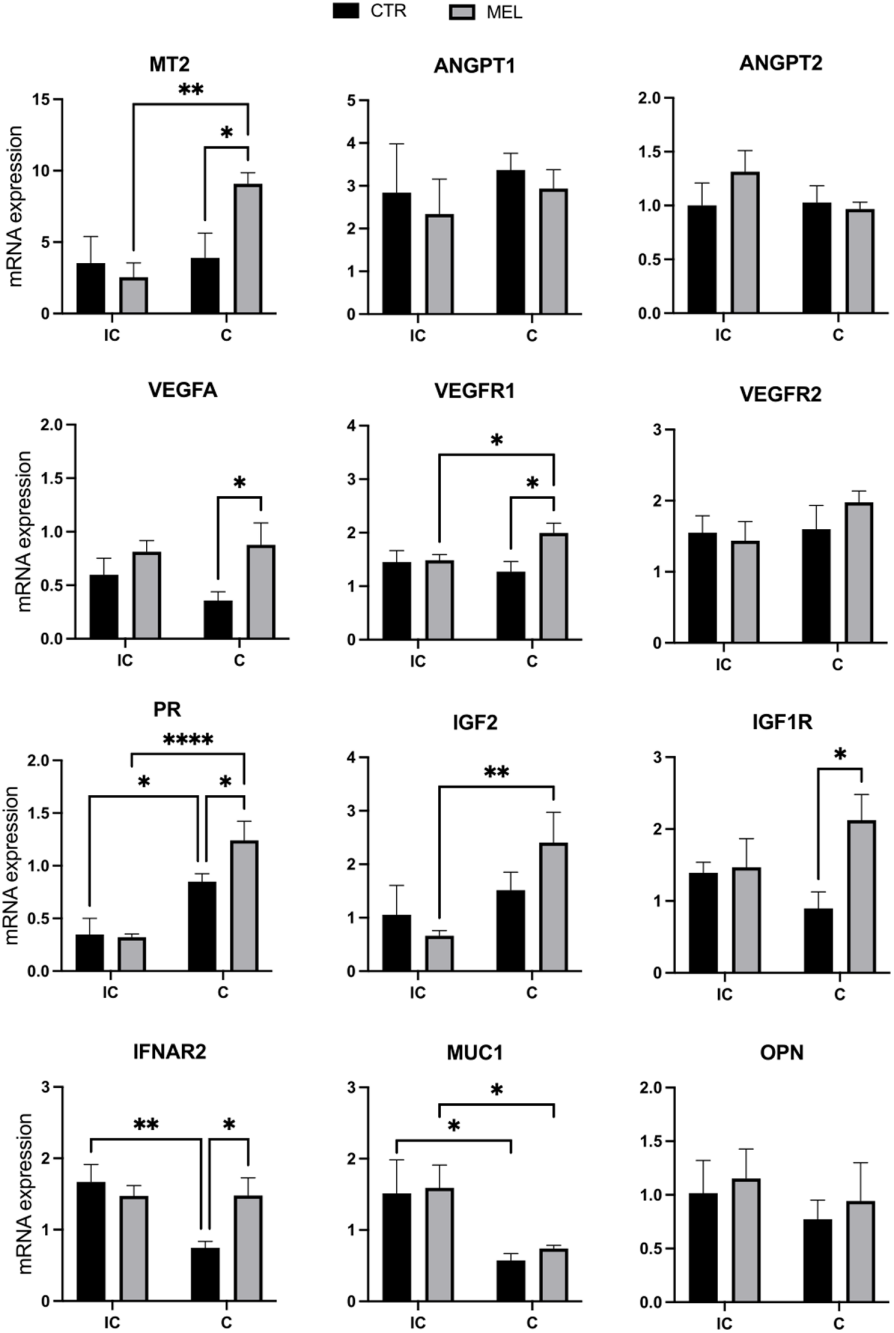
Gene expression profile of endometrium. Relative mRNA expression levels of angiogenic and developmentally relevant factors were determined in the intercaruncular (IC) and caruncular (C) endometrium of day 21 pregnant sheep implanted (MEL) or not (CTR) with melatonin for 50 days. Expression levels of the target genes were normalized to β-actin (*ACTB*) and *GAPDH* as internal controls. Data are reported as mean arbitrary units ± s.e.m. (*P < 0.05; **P < 0.01; ****P < 0.0001).

Melatonin treatment increased significantly (*P* < 0.05) the expression of the *VEGFA* and *VEGFR1* genes but did not affect the expression of the other genes evaluated. Nevertheless, the differences between groups indicated that the effect was restricted to the caruncular region. Similarly, exogenous melatonin increased the expression of the *PR*, *IFNAR2*, *IGF1R*, and *MT2* genes (*P* < 0.05, Fig. 3) in the caruncles but did not have a significant effect on gene expression in the intercaruncular endometrium.

Overall, the expression of *IGF2* and *MT2* was significantly (*P* < 0.05) higher and expression of *IFNAR2* (*P* < 0.05) and that of *MUC1* (*P* < 0.01) were lower in the caruncular region than they were in the intercaruncular region; however, the increase in the expression of *IGF2* and *MT2* in the caruncular region was observed only in the MEL ewes, and the same effect was observed for *VEGFR1* (*P* < 0.01). The expression of *PR* was significantly higher in the caruncles of both the CTR (*P* < 0.0001) and MEL (*P* < 0.05) ewes than it was in the intercaruncular region. In both MEL and CTR ewes, the expression of *MUC1* was significantly (*P* < 0.05) lower in the caruncles. Expression of *IFNAR2* was significantly (P < 0.01) lower in the caruncles than it was in the intercaruncular regions in the CTR but not in the MEL ewes.

### Placenta histology, gene and protein expression profiles

**Figure 4:**
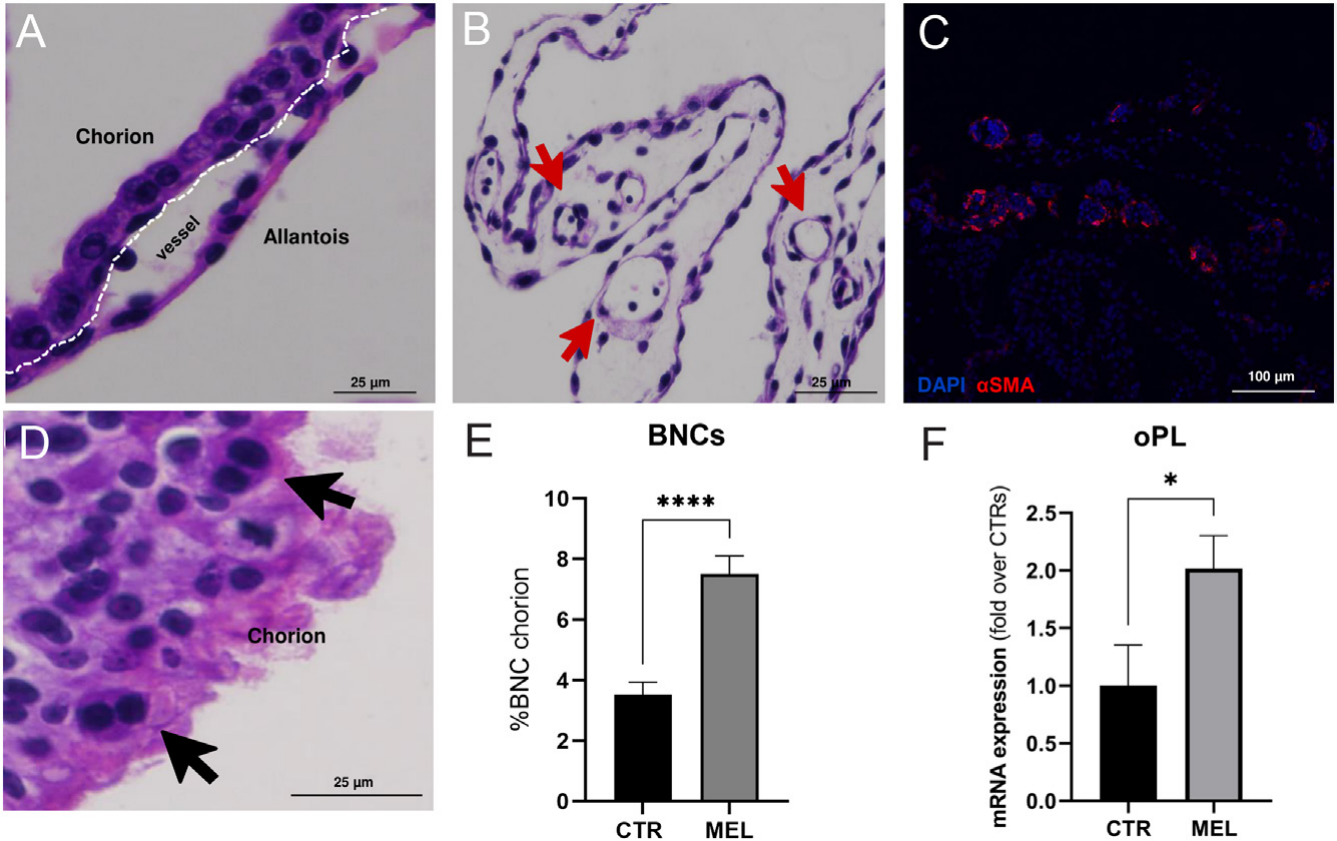
Morphology, BNCs detection, and *oPL* expression in the placenta. The figure panel shows the morphology of chorion-allantois and vessels of the placentas in ewes that received a melatonin implant. (A) Placenta had a chorion that was mainly composed of mononuclear cuboidal-shaped cells in a well-organized layer that was juxtaposed to the allantoic tissue. (B,C) Early blood vessels are identified in the allantoic region (red arrows), where aSMA detection (red staining) revealed the vessels’ structure. (C) The photo on the bottom left shows an example of BNCs in the chorion layer (black arrows). (D,E) Placentas from MEL ewes had many more BNCs than did those from CTR ewes, and had a higher *oPL* expression. Data are reported as mean ± s.e.m. for BNC counts and *oPL* mRNA expression, respectively (*P < 0.05; ****P < 0.0001).

**Figure 5:**
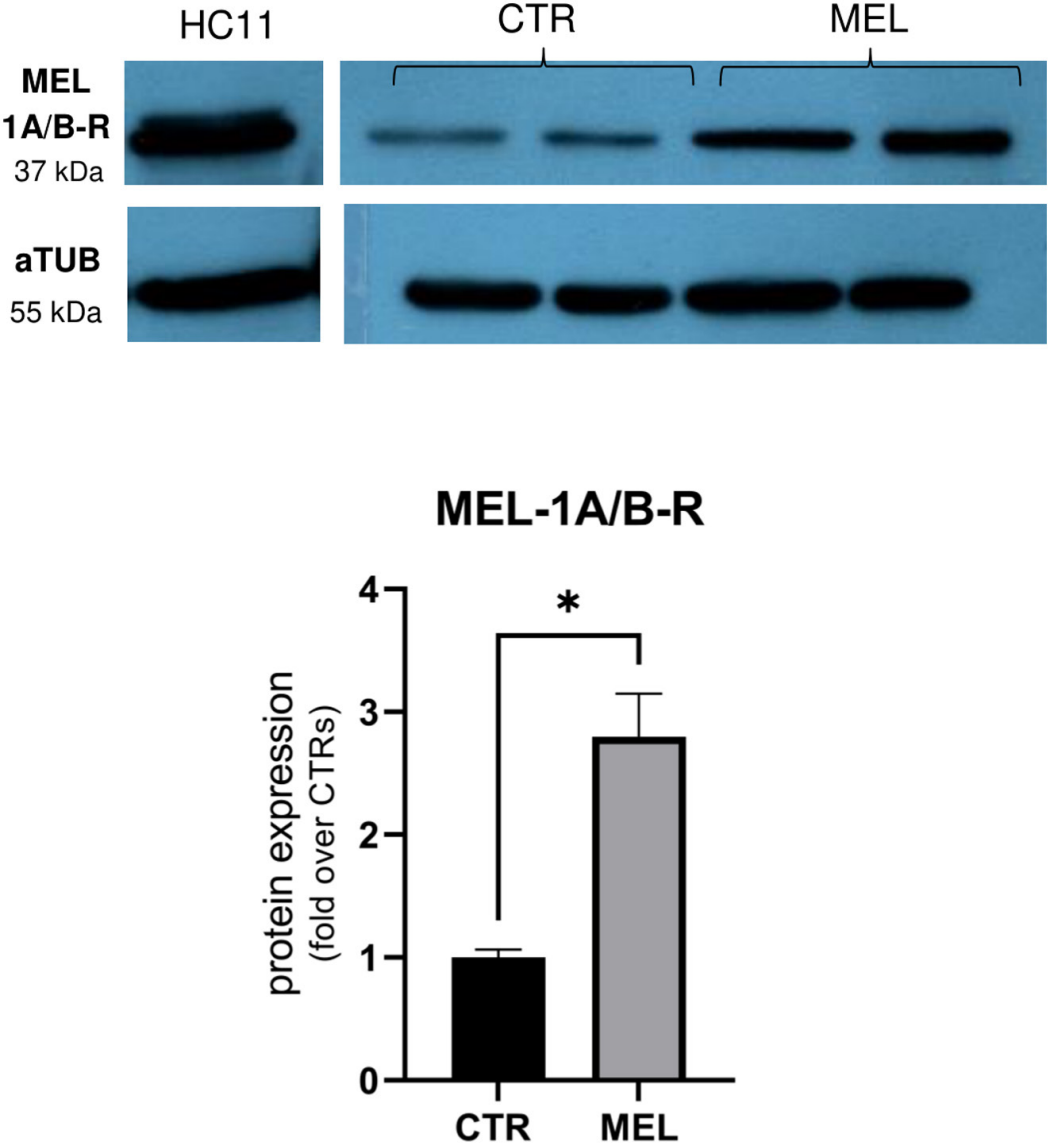
Melatonin receptor detection in the placenta of sheep in early pregnancy. Western blot analysis revealed that the expression of melatonin receptors in the placenta was significantly higher in melatonin-treated ewes than it was in non-implanted ewes. The HC11 cell line was used as a positive control and aTUB was the reference protein. Densitometry is expressed as a mean ± s.d. (*P < 0.05).

**Figure 6:**
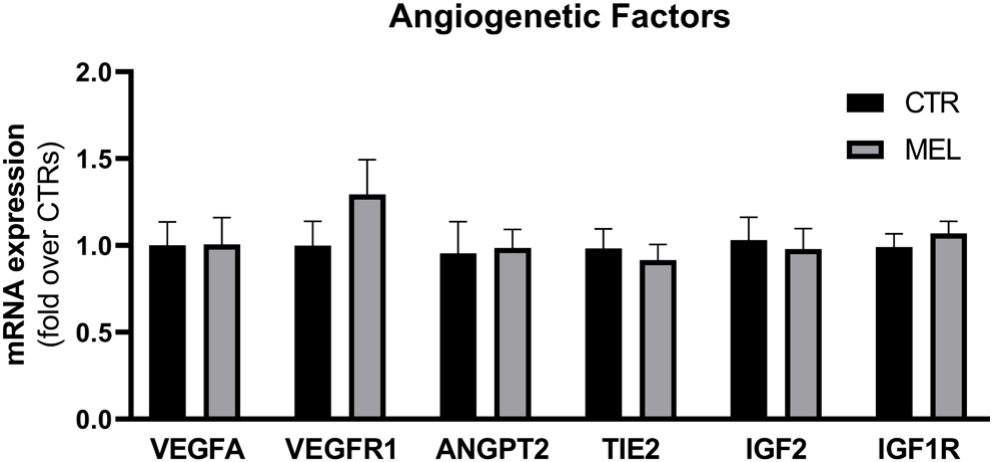
Gene expression profiles in the placenta. A panel of angiogenic factors involved in early placenta development was selected and their mRNA expression was studied in ewes that either did (MEL) or did not (CTR) receive a melatonin implant. Data are reported as mean ± s.e.m., and groups did not differ significantly.

Chorion-allantoid tissues of CTR and MEL placentas had similar morphologies at day 21 of pregnancy (Fig. 4). The juxtaposition of chorion and allantois layers in the placentas of control and melatonin-treated ewes was normal (Fig. 4A). At the chorion level, we observed cuboid-shaped cells that contained a single nucleus and binucleated cells (BNC) that were round and had more eosinophilic cytoplasm (Fig. 4D). The number of BNC in the trophectoderm epithelium was significantly (*P* < 0.0001) higher in the placentas of the MEL group than it was in the CTR group (Fig. 4E). In addition, a similarly developing vascular network was present in the allantoic regions in both groups (Fig. 4B). Immunohistochemistry analysis confirmed the expression of aSMA, a vessel maturation marker (Fig. 4C). Western blot analysis of melatonin receptors in the placenta indicated that expression was significantly (*P* < 0.05) higher in the MEL group than it was in the CTR group (Fig. 5). Analyses of gene expression in CTR and MEL placentas revealed that melatonin supplementation upregulates *oPL* mRNA expression (*P* = 0.04) (Fig. 4F); however, the expression of angiogenic factors (*VEGFA*, *VEGFR1*, *IGF2*, *IGF1R*, *ANGPT2*, and *TIE2*) did not differ significantly between groups (Fig. 6).

## Discussion

This study explored the effects of exogenous melatonin on the uterine microenvironment in the early stages of pregnancy in sheep. Exogenous melatonin supplementation influenced the endometrial and placental response for supporting embryo implantation and survival. In addition, melatonin acted on multiple mechanisms involved in pregnancy maintenance in the peri-implantation period. Melatonin treatment increased the number of embryos per ewe collected on day 21 of pregnancy, and earlier studies reported that melatonin had a beneficial role in *in vitro* blastocyst maturation and embryo development in sheep (Buffoni *et al.* 2014, Voiculescu *et al.* 2014). Although it cannot be known whether all embryos in our study would have developed to term, an increase in average litter size has been reported in melatonin-treated sheep (Abecia *et al.* 2007). Therefore, we suspected that melatonin supplements in the peri-implantation period might improve pregnancy outcomes in sheep. In support of that hypothesis, the range in sizes of embryos was narrower in melatonin-implanted ewes than in control ewes, which suggests a synchronization effect of melatonin on embryo development in early pregnancy, when most of the pregnancy losses occur in small ruminants (Diskin & Morris 2008). In addition, P4 action at the uterine level influences the embryo survival rate (Spencer *et al.* 2004). In our study, melatonin implants increased P4 plasma concentrations in ewes, as reported by Forcada *et al.* (2006), underscoring the possible role of melatonin in maintaining corpus luteum activity and, therefore, P4 secretion in sheep. In addition, exogenous melatonin increased the release of b-HCG in trophoblast cells in humans (Soliman *et al.* 2015). Recently, Duan *et al.* (2024) reported that melatonin promotes ovary activity by stimulating P4 secretion in sheep. In our study, the effect of melatonin on the P4 signaling system axis was indicated by an increase in PR in the caruncles of MEL ewes. In another study, melatonin treatment increased the abundance of PR on day 5 of pregnancy in the deep endometrial glands in sheep (Vázquez *et al.* 2013), and a similar effect was observed in the uterus of melatonin-treated rats (Abd-Allah *et al.* 2003). P4 concentration is influenced by the number of corpora lutea; nevertheless, our study has shown that MEL ewes had higher P4 levels than did CTR ewes that had similar numbers of fetuses (2.7 vs 3.1 ng/mL). In early pregnancy in sheep, the ovarian P4 supply must be maintained until about day 50, when the placenta starts to produce this hormone (Ricketts & Flint 1980). In our study, exogenous melatonin increased the caruncular gene expression of *IFNAR2*, which suggests a greater sensitivity to the embryo antiluteolytic signal, the interferon tau (IFNT). In addition to reinforcing the luteoprotective effect of IFNT, it facilitates the expression of interferon-stimulated genes that are essential for uterine receptivity to implantation (Bazer *et al.* 2008).

In examining the uterine molecular response, we observed that melatonin treatment doubled the expression of the MT2 receptor in the caruncles, which demonstrated a region-specific melatonin signaling activation. The caruncle represents the maternal portion of the placentome (sheep placenta morpho-functional unit; Davenport *et al.* 2023), which suggests that melatonin might affect fetal–maternal cross-talk. In addition, the conceptus produces IGFs, and the IGF2–IGF1R ligand–receptor pair is one of the most prominently expressed in the extraembryonic membrane–endometrium interface in sheep (Jia *et al.* 2023). Like Stevenson *et al.* (1994), we found that *IGF2* mRNA was more strongly expressed in the caruncles than it was in the intercaruncular endometrium. Furthermore, the melatonin implant increased the caruncular expression of *IGF1R*, which suggests that the hormone supplement increased cross-talk in pregnant sheep.

Among angiogenic factors, which are responsible for ensuring adequate nutrient delivery, exogenous melatonin increased the expression of *VEGFA* and its receptor *VEGFR1* in the caruncular region. In sheep, the increase in VEGF precedes uterine growth and the development of the microvasculature, and it is associated with an increase in the capillary size in the caruncle (Redmer *et al.* 1998, Reynolds *et al.* 2010). Possibly, the increase in endometrial expression observed in MEL ewes in our study might have enhanced maternal nutrient supply because of an improvement in caruncular vessel development.

In our study, the expression of the antiadhesive *MUC1* was much lower in the caruncles than it was in the surrounding endometrium, but *OPN* remained high in both regions, which is consistent with the known roles that these glycoproteins play in the implantation cascade in sheep (Johnson *et al.* 2001, 2014). Exogenous melatonin did not have a significant effect on *MUC1* or *OPN*. Effects of melatonin on endometrial extracellular matrix adhesion cannot be discarded since other relevant molecules, such as galectin 15 and integrin subunits, could be regulated.

Given the uterine physiological responses to exogenous melatonin, we examined the effects of melatonin on placental development and the expression of developmentally important factors. Studies have shown that the placenta expresses the melatonin machinery throughout pregnancy in humans (Lanoix *et al.* 2008, Soliman *et al.* 2015), but published data on sheep are available for mid-to-late pregnancy only (Eifert *et al.* 2015, Lemley & Vonnahme 2017, Sales *et al.* 2018). Our study has revealed that melatonin receptors (MT1/MT2) in the placenta are expressed in early pregnancy in sheep. Furthermore, the increase in placental lactogen (oPL) expression observed in the MEL ewes reflected an increase in the activation of melatonin signaling in these sheep. Exogenous melatonin did not appear to adversely affect chorion-allantoic development. An *in vitro* experiment by Ma *et al. (*2020) demonstrated that melatonin exerts pro- and anti-vascularization actions depending on the physiological and pathological conditions; therefore, we investigated whether melatonin improves vessel development and the expression of angiogenic factors. In our study, the vascular architecture did not differ significantly between treatment and control ewes. Furthermore, the immunolocalization of aSMA, which is associated with normal pericyte maturation on the basement membrane of the vascular endothelium (Benjamin *et al.* 1998), was similar in both groups. In addition, the molecular analyses indicated that the mRNA expression of angiogenic factors in the two groups was similar. Other sheep studies reported that under adverse conditions, such as intrauterine growth restriction (Lemley *et al.* 2012) or maternal undernutrition (Eifert *et al.* 2015), melatonin supplementation increased umbilical blood flow by approximately 20%; however, in mid-to-late gestation there was no significant effect on placental vascularity. In this light, we surmise that the beneficial effect of exogenous melatonin emerges when the conceptus is forced to grow in a suboptimal environment.

In our study, the positive effect of melatonin might have been related to the increase in BNCs, which are a feature of trophoblast differentiation in ruminants (Wooding 1984, 2022). BNCs migrate from the chorion layer and fuse with the maternal epithelium to ensure embryo implantation (Spencer *et al.* 2004). Our results suggest that melatonin supports the rearrangement of the fetal–maternal interface in early pregnancy. In another study, melatonin did not affect BNC differentiation in mid-to-late gestation in ewes (Mansour *et al.* 2019). Possibly, melatonin plays a role in placenta development in a time-dependent manner (Vasquez-Hidalgo *et al.* 2023). In the sheep placenta, BNCs exert most of the endocrine activity by releasing P4 and oPL (Spencer *et al.* 2004, Seo *et al.* 2019). Our study showed an increase in oPL mRNA expression in the placentas of MEL ewes. Takahashi *et al.* (2013) reported that oPL acted in partitioning nutrients in undernourished cows, and, in another study, plasma placental lactogen levels were lowest in women who were affected by placental insufficiency in which melatonin levels appeared to be reduced (Berbets *et al.* 2021). In general, we suspect that melatonin indirectly favors placenta efficiency in sheep.

In conclusion, our study demonstrated the effect of melatonin on the endometrium and placenta in early pregnancy in sheep. An increase in maternal plasma progesterone in melatonin-implanted ewes fostered uterine receptivity to embryo implantation. Melatonin supports pregnancy by acting on both sides of the fetal–maternal interface. At the uterine level, it acts in a region-specific manner, mainly improving the caruncular expression of developmentally crucial genes; in the placenta, it influences binuclear cell differentiation. Therefore, exogenous melatonin contributes to creating a more favorable uterine microenvironment for proper conceptus development and pregnancy maintenance.

## Declaration of interest

The authors declare that there is no conflict of interest that could be perceived as prejudicing the impartiality of the research reported.

## Funding

This research was supported by a Grant for Internationalization from the University of Turin, Italy (TOSP_GFI_22_01_F), Fondazione CRThttp://dx.doi.org/10.13039/100007364 (TOSP_CRT_22_01), and the Gobierno de Aragón (group BIOFITER). FC was funded by the National Agency for Research and Developmenthttp://dx.doi.org/10.13039/100006190/Scholarship Program/Doctorado Becas Chile/2020 – 72210031.

## Author contributions statement

IV, JA, and PT conceived and planned the study. IV, FC, PA, SM, and JA managed the animals. IV, CS, PA, IM, FC, and PT performed the laboratory analyses. IV, FC, PA, CS, SM, IM, JA, and PT performed data analyses and interpretation. IV, CS, JA, and PT conducted performed a literature search and wrote the paper. JA and PT supervised the project and provided funding. All authors reviewed the results and approved the final version of the manuscript.

## References

[bib1] Abd-Allah ARA El-Sayed el SM Abdel-Wahab MH & Hamada FMA 2003 Effect of melatonin on estrogen and progesterone receptors in relation to uterine contraction in rats. Pharmacological Research 47 349–354. (10.1016/s1043-6618(0300014-8) 12644393

[bib4] Abecia JA Valares JA Forcada F Palacín I Martín S & Martino A 2007 The effect of melatonin on the reproductive performance of three sheep breeds in Spain. Small Ruminant Research 69 10–16. (10.1016/j.smallrumres.2005.12.018)

[bib2] Abecia JA Forcada F Casao A & Palacín I 2008 Effect of exogenous melatonin on the ovary, the embryo and the establishment of pregnancy in sheep. Animal 2 399–404. (10.1017/S1751731107001383) 22445042

[bib3] Abecia JA Forcada F Vázquez MI Muiño-Blanco T Cebrián-Pérez JA Pérez-Pe R & Casao A 2018 Role of melatonin on embryo viability in sheep. Reproduction, Fertility, and Development 31 82–92. (10.1071/RD18308) 32188544

[bib5] Bazer FW Burghardt RC Johnson GA Spencer TE & Wu G 2008 Interferons and progesterone for establishment and maintenance of pregnancy: interactions among novel cell signaling pathways. Reproductive Biology 8 179–211. (10.1016/s1642-431x(1260012-6) 19092983

[bib6] Benjamin LE Hemo I & Keshet E 1998 A plasticity window for blood vessel remodelling is defined by pericyte coverage of the preformed endothelial network and is regulated by PDGF-B and VEGF. Development 125 1591–1598. (10.1242/dev.125.9.1591) 9521897

[bib7] Berbets A Koval H Barbe A Albota O & Yuzko O 2021 Melatonin decreases and cytokines increase in women with placental insufficiency. Journal of Maternal-Fetal and Neonatal Medicine 34 373–378. (10.1080/14767058.2019.1608432) 31023180

[bib8] Buffoni A Vozzi PA Gonzalez DM Rios G Viegas-Bordeira H & Abecia JA 2014 The effect of melatonin and season on in vivo embryo production of Dohne Merino ewes. Small Ruminant Research 120 121–124. (10.1016/j.smallrumres.2014.05.003)

[bib151] Butler H & Juurlink BHJ 1987 Correlation graphs. In An Atlas for Staging Mammalian and Chick Embryos. 1st edn, pp.189–206. Boca Raton, Florida, USA: CRC Press. (10.1201/9781351069939)

[bib9] Casao A Pérez-Pé R Cebrián-Pérez JA Muiño-Blanco T Forcada F & Abecia JA 2019 Presence of melatonin receptors in ovine blastocysts. Reproduction, Fertility and Development 31 188. (10.1071/RDv31n1Ab125)

[bib10] Cosso G Mura MC Pulinas L Curone G Vigo D Carcangiu V & Luridiana S 2021 Effects of melatonin treatment on milk traits, reproductive performance and immune response in Sarda dairy sheep. Italian Journal of Animal Science 20 632–639. (10.1080/1828051X.2021.1904796)

[bib11] Davenport KM Ortega MS Johnson GA Seo H & Spencer TE 2023 Review: implantation and placentation in ruminants. Animal 17(Supplement 1) 100796. (10.1016/j.animal.2023.100796) 37567669

[bib12] De la Concha-Bermejillo A & Romano J 2021 Pregnancy loss in ruminants. Clinical Theriogenology 13 181–193. (10.58292/ct.v13.9335)

[bib13] Diskin MG & Morris DG 2008 Embryonic and early foetal losses in cattle and other ruminants. Reproduction in Domestic Animals 43(Supplement 2) 260–267. (10.1111/j.1439-0531.2008.01171.x) 18638133

[bib152] Duan H, Yang S, Xiao L, Yang S, Yan Z, Wang F, Ma X, Zhang L, Zhang Y, Hu J Zhao X 2024 Melatonin promotes progesterone secretion in sheep luteal cells by regulating autophagy via the AMPK/mTOR pathway. Theriogenology 214 342–351. (https://doi:) 37976799 10.1016/j.theriogenology.2023.11.010

[bib14] Eifert AW Wilson ME Vonnahme KA Camacho LE Borowicz PP Redmer DA Romero S Dorsam S Haring J & Lemley CO 2015 Effect of melatonin or maternal nutrient restriction on vascularity and cell proliferation in the ovine placenta. Animal Reproduction Science 153 13–21. (10.1016/j.anireprosci.2014.11.022) 25578503

[bib15] Forcada F Abecia JA Cebrián-Pérez JA Muiño-Blanco T Valares JA Palacín I & Casao A 2006 The effect of melatonin implants during the seasonal anestrus on embryo production after superovulation in aged high-prolificacy Rasa Aragonesa ewes. Theriogenology 65 356–365. (10.1016/j.theriogenology.2005.05.038) 15967490

[bib16] González-Arto M Aguilar D Gaspar-Torrubia E Gallego M Carvajal-Serna M Herrera-Marcos LV Serrano-Blesa E Hamilton TR Pérez-Pé R Muiño-Blanco T, *et al.* 2017 Melatonin MT_1_ and MT_2_ receptors in the ram reproductive tract. International Journal of Molecular Sciences 18 662. (10.3390/ijms18030662) 28335493 PMC5372674

[bib17] Jia GX Ma WJ Wu ZB Li S Zhang XQ He Z Wu SX Tao HP Fang Y Song YW, *et al.* 2023 Single-cell transcriptomic characterization of sheep conceptus elongation and implantation. Cell Reports 42 112860. (10.1016/j.celrep.2023.112860) 37494181

[bib18] Johnson GA Bazer FW Jaeger LA Ka H Garlow JE Pfarrer C Spencer TE & Burghardt RC 2001 Muc-1, integrin, and osteopontin expression during the implantation cascade in sheep. Biology of Reproduction 65 820–828. (10.1095/biolreprod65.3.820) 11514347

[bib19] Johnson GA Burghardt RC & Bazer FW 2014 Osteopontin: a leading candidate adhesion molecule for implantation in pigs and sheep. Journal of Animal Science and Biotechnology 5 56. (10.1186/2049-1891-5-56) 25671104 PMC4322467

[bib20] Kaufman MH 1995 Methodology. In The Atlas of Mouse Development, rev ed. London: Eds Elsevier Academic Press, pp. 2–5.

[bib21] Lanoix D Beghdadi H Lafond J & Vaillancourt C 2008 Human placental trophoblasts synthesize melatonin and express its receptors. Journal of Pineal Research 45 50–60. (10.1111/j.1600-079X.2008.00555.x) 18312298

[bib23] Lemley CO & Vonnahme KA 2017 Physiology and endocrinology symposium: alterations in uteroplacental hemodynamics during melatonin supplementation in sheep and cattle. Journal of Animal Science 95 2211–2221. (10.2527/jas.2016.1151) 28726984

[bib22] Lemley CO Meyer AM Camacho LE Neville TL Newman DJ Caton JS & Vonnahme KA 2012 Melatonin supplementation alters uteroplacental hemodynamics and fetal development in an ovine model of intrauterine growth restriction. American Journal of Physiology. Regulatory, Integrative and Comparative Physiology 302 R454–R467. (10.1152/ajpregu.00407.2011) 22129617

[bib24] Ma Q Reiter RJ & Chen Y 2020 Role of melatonin in controlling angiogenesis under physiological and pathological conditions. Angiogenesis 23 91–104. (10.1007/s10456-019-09689-7) 31650428

[bib25] Mansour H Lemley CO Anthony R Swanson KC Grazul-Bilska AT & Mansour H 2019 PSII-35 melatonin supplementation and restricted nutrition do not affect chorionic somatomammotropin (CSH) concentration in ovine placenta from mid- to late- gestation. Journal of Animal Science 97(3) 244. (10.1093/jas/skz258.496)

[bib26] McCarty KJ Owen MPT Hart CG Thompson RC Burnett DD King EH Hopper RM & Lemley CO 2018 Effect of chronic melatonin supplementation during mid to late gestation on maternal uterine artery blood flow and subsequent development of male offspring in beef cattle. Journal of Animal Science 96 5100–5111. (10.1093/jas/sky363) 30203092 PMC6276587

[bib150] Presman DM, Hoijman E, Ceballos NR, Galigniana MR & Pecci A 2006 Melatonin inhibits glucocorticoid receptor nuclear translocation in mouse thymocytes. Endocrinology 147 5452–5459. (10.1210/en.2006-0252) 16916958

[bib27] Ptak GE D'Agostino A Toschi P Fidanza A Zacchini F Czernik M Monaco F & Loi P 2013 Post-implantation mortality of in vitro produced embryos is associated with DNA methyltransferase 1 dysfunction in sheep placenta. Human Reproduction 28 298–305. (10.1093/humrep/des397) 23169866

[bib28] Redmer DA Kraft KC Kirsch JD & Reynolds LP 1998 Expression and localization of vascular endothelial growth factor (VEGF) in ovine placenta during late pregnancy. Biology of Reproduction 58 202.

[bib29] Reiter RJ Tan DX Manchester LC Paredes SD Mayo JC & Sainz RM 2009 Melatonin and reproduction revisited. Biology of Reproduction 81 445–456. (10.1095/biolreprod.108.075655) 19439728

[bib30] Reynolds LP Borowicz PP Caton JS Vonnahme KA Luther JS Hammer CJ Maddock Carlin KR Grazul-Bilska AT & Redmer DA 2010 Developmental programming: the concept, large animal models, and the key role of uteroplacental vascular development. Journal of Animal Science 88 E61–E72. (10.2527/jas.2009-2359) 20023136

[bib31] Ricketts AP & Flint AP 1980 Onset of synthesis of progesterone by ovine placenta. Journal of Endocrinology 86 337–347. (10.1677/joe.0.0860337) 6933207

[bib32] Roberts RM Green JA & Schulz LC 2016 The evolution of the placenta. Reproduction 152 R179–R189. (10.1530/REP-16-0325) 27486265 PMC5033709

[bib33] Sales F Parraguez V McCoard S Cofré E Peralta O Subiabre I & González-Bulnes A 2018 PSXV-19 fetal blood oxygenation and placental efficiency is increased by melatonin implants in sheep. Journal of Animal Science 96(Supplement 3) 239–240. (10.1093/jas/sky404.522)

[bib34] Seo H Bazer FW Burghardt RC & Johnson GA 2019 Immunohistochemical examination of trophoblast syncytialization during early placentation in sheep. International Journal of Molecular Sciences 20 4530. (10.3390/ijms20184530) 31540219 PMC6769582

[bib35] Soliman A Lacasse AA Lanoix D Sagrillo-Fagundes L Boulard V & Vaillancourt C 2015 Placental melatonin system is present throughout pregnancy and regulates villous trophoblast differentiation. Journal of Pineal Research 59 38–46. (10.1111/jpi.12236) 25833399

[bib36] Sosa C Laurenzana E de Brun V Meikle A & Abecia JA 2023 The melatonin system is expressed in the ovine uterus: effect of the day of the oestrous cycle and undernutrition. Reproduction, Fertility, and Development 35 563–574. (10.1071/RD22194) 37290449

[bib37] Spencer TE Johnson GA Burghardt RC & Bazer FW 2004 Progesterone and placental hormone actions on the uterus: insights from domestic animals. Biology of Reproduction 71 2–10. (10.1095/biolreprod.103.024133) 14973264

[bib38] Stevenson KR Gilmour RS & Wathes DC 1994 Localization of insulin-like growth factor-I (IGF-I) and -II messenger ribonucleic acid and type 1 IGF receptors in the ovine uterus during the estrous cycle and early pregnancy. Endocrinology 134 1655–1664. (10.1210/endo.134.4.8137728) 8137728

[bib39] Takahashi T Hayashi KG & Hosoe M 2013 Biology of the placental proteins in domestic ruminants: expression, proposed roles and practical applications. Japan Agricultural Research Quarterly 47 43–51. (10.6090/jarq.47.43)

[bib40] Torres-Farfan C Valenzuela FJ Mondaca M Valenzuela GJ Krause B Herrera EA Riquelme R Llanos AJ & Seron-Ferre M 2008 Evidence of a role for melatonin in fetal sheep physiology: direct actions of melatonin on fetal cerebral artery, brown adipose tissue and adrenal gland. Journal of Physiology 586 4017–4027. (10.1113/jphysiol.2008.154351) 18599539 PMC2538916

[bib41] Trotta RJ Lemley CO Vonnahme KA & Swanson KC 2021 Effects of nutrient restriction and melatonin supplementation from mid-to-late gestation on maternal and fetal small intestinal carbohydrase activities in sheep. Domestic Animal Endocrinology 74 106555. (10.1016/j.domaniend.2020.106555) 32947201

[bib42] Vasquez-Hidalgo MA Grazul-Bilska AT Swanson KC Perry GA & Vonnahme KA 2023 Timing and duration of nutrient restriction and its impacts on placental development and umbilical blood flow in adolescent sheep. Theriogenology 209 21–30. (10.1016/j.theriogenology.2023.06.016) 37354757

[bib43] Vázquez MI Forcada F Sosa C Casao A Sartore I Fernández-Foren A Meikle A & Abecia JA 2013 Effect of exogenous melatonin on embryo viability and uterine environment in undernourished ewes. Animal Reproduction Science 141 52–61. (10.1016/j.anireprosci.2013.07.007) 23948208

[bib44] Viola I Toschi P Manenti I Accornero P & Baratta M 2023 Modulatory role of mTOR in trophoblast adaptive response in the early stage of placentation in sheep. Reproduction 165 313–324. (10.1530/REP-22-0356) 36602917

[bib45] Voiculescu SE Zygouropoulos N Zahiu CD & Zagrean AM 2014 Role of melatonin in embryo fetal development. Journal of Medicine and Life 7 488–492. 25713608 PMC4316124

[bib46] Wooding FBP 1984 Role of binucleate cells in fetomaternal cell fusion at implantation in the sheep. American Journal of Anatomy 170 233–250. (10.1002/aja.1001700208) 6465051

[bib47] Wooding FBP 2022 The ruminant placental trophoblast binucleate cell: an evolutionary breakthrough. Biology of Reproduction 107 705–716. (10.1093/biolre/ioac107) 35594454 PMC9476219

